# Parental non-hereditary teratogenic exposure factors on the occurrence of congenital heart disease in the offspring in the northeastern Sichuan, China

**DOI:** 10.1038/s41598-020-60798-6

**Published:** 2020-03-03

**Authors:** Yun Liang, Xingsheng Hu, Xiaoqin Li, Bing Wen, Liang Wang, Cheng Wang

**Affiliations:** 1 Department of Pediatric Surgery, Affiliated Hospital of North Sichuan Medical College, Nanchong, 637000 P.R. China; 2 Department of Oncology, the Second Xiangya Hospital of Central South University, Changsha, Hunan 41011 P.R. China; 3 Department of Nursing, Affiliated Hospital of North Sichuan Medical College, Nanchong, 637000 P.R. China; 4 Department of Cardiothoracic Surgery, Nanchong Central Hospital, Nanchong, 637000 P.R. China

**Keywords:** Cardiac device therapy, Cardiac device therapy, Interventional cardiology, Interventional cardiology, Congenital heart defects

## Abstract

Nonhereditary factors play an important role in the occurrence of congenital heart disease (CHD). This study was to explore the possible parental nonhereditary exposure factors relevant to the occurrence of CHD in the northeastern Sichuan area. A total of 367 children with CHD and 367 children without congenital malformations aged 0 to 14 years old were recruited from the Affiliated Hospital of North Sichuan Medical College and Nanchong Central Hospital between March 2016 and November 2018. This study was designed as a case-control study with 1:1 frequency matching, in which the parents of cases and controls were interviewed with the same questionnaire according to the gestational age of the child, maternal age during pregnancy and the same maternal race/ethnicity. Then, 322 matched case-control pairs were analysed by SPSS 22. Thirty-one suspicious factors were entered into the binary logistic regression analysis after univariate regression analysis of 55 factors (alpha = 0.05). The analysis results showed that 7 factors were significantly associated with the occurrence of CHD. Thus, augmenting maternal mental healthcare, improving the quality of drinking water, obtaining adequate nutrition, maintaining a healthy physical condition during pregnancy, enhancing parents’ level of knowledge and maintaining a healthy lifestyle may lower the occurrence of CHD.

## Introduction

Congenital heart disease (CHD) is a multifactorial disease that is caused by genetic and nonhereditary factors, resulting in abnormalities in cardiovascular development^1^. In China, more than 30,000 children with CHD are born annually; this is the most common birth defect among infants^2^. Nonhereditary factors play an important role in the occurrence of CHD, including physical, chemical, biological, psychological and other factors^3^. Because the living environment and lifestyle of people in different areas are quite different, it is of great clinical and epidemiological significance to study the main nonhereditary risk factors of CHD in specific areas.

Northeastern Sichuan is a poverty-stricken area in China, and agriculture is its main economic pillar^4^. In addition, northeastern Sichuan is a hilly area that is not suitable for the use of modern agricultural machinery and equipment^5^. To improve crop yield, chemical fertilizer is widely used^6^. Therefore, in the process of agricultural production, people have more direct contact with pesticides and other harmful substances^6–8^. In addition, some non-standard small factories directly discharge factory exhaust gas and wastewater to the environment^9,10^. The groundwater of residents is polluted by industrial and agricultural wastewater^11,12^. Smoking and drinking are currently common unhealthy habits among young people^13^. Teenagers’ sexual lives are casual and lack the consciousness of pregnancy prevention and prenatal examination^14,15^. Therefore, unintended pregnancy has become a common phenomenon^16^. All of the above factors may affect the occurrence of CHD^17^. The purpose of this study was to explore the possible parental nonhereditary exposure factors relevant to the occurrence of CHD in the northeastern Sichuan area, evaluate the relative importance of each risk factor, and then provide scientific guidelines for CHD prevention.

## Results

### Composition of the object of study

The 322 matched case-control pairs were analysed by SPSS from the original cases in the study population (Tables 1 and 2). Among the 322 analysed cases, 182 (56.5%) were males and 140 (43.5%) were females. There were 163 (50.6%) males and 159 (49.4%) females in the matched control group. There was no significant difference in the gender distribution between the two groups (χ^2^ = 2.254, P = 0.133) (Table 3). The distribution of children in different age groups in the two groups is shown in Table 3, and the difference was not statistically significant (χ^2^ = 4.826, P = 0.566).

**Table 1:** Case-Control Matching Statistics.

Match Type	Count
Exact Matches	124
Fuzzy Matches	198
Unmatched Including Missing Keys	45
Unmatched With Valid Keys	45
Sampling	Without Replacement
Log File	None
Maximize Matching Performance	Yes

**Table 2:** Case-Control Match Tolerances.

Match Variables	Value	Fuzzy Match Attempts	Incremental Rejection Percentage
Exact (All Variables)		8497.000	98.541
Gestational Age	0.000	8373.000	49.039
Maternal Racial/Ethnic	0.000	4267.000	40.005
Maternal Age of Pregnancy	1.000	2560.000	92.266

**Table 3:** Gender and Age Distribution of Children.

	Cases (n = 322)		Controls (n = 322)		χ^2^ value	P Value
	N	%	N	%		
Gender					2.254	0.133
Male	182	56.5	163	50.6		
Female	140	43.5	159	49.4		
Age (years*)					4.826	0.566
~1	79	24.5	96	29.8		
~2	61	19.9	63	19.6		
~3	44	13.7	42	13.0		
~4	22	6.8	26	8.1		
~5	17	5.3	14	4.3		
~6	22	6.8	14	4.3		
Above 6	77	23.9	67	20.8		

### Univariate analysis of research factors among cases and controls

Thirty-one factors were statistically significant after univariate conditional logistic regression analysis of all 55 research factors (alpha = 0.05) (Supplementary Table 1).

### Multivariate analysis

Thirty-one candidate factors entered the analysis of binary logistic regression by the forward likelihood ratio method (significance level to enter = 0.05, significance level to remain = 0.10). The analysis results showed that 7 factors were significantly associated with the occurrence of CHD: maternal adverse emotions during pregnancy (OR = 2.650, 95% CI: 1.903~3.692), maternal exposure to polluted water (OR = 2.205, 95% CI: 1.240~3.921), paternal active smoking (OR = 1.967, 95% CI: 1.357~2.850), maternal severe vomiting (OR = 1.911, 95% CI: 1.067~3.424), maternal chronic diseases during pregnancy (OR = 1.852, 95% CI: 1.012~3.387), maternal nutrition supplementation during pregnancy (OR = 0.588, 95% CI: 0.412~0.841), and paternal education level (OR = 0.585, 95% CI: 0.405~0.845) (Table 4). As shown in Table 4, the occurrence of CHD was not associated with paternal heavy metal exposure factors (OR = 1.990, 95% CI: 0.999~3.963), although P  =  0.05. The protective factors to prevent CHD were maternal nutrition supplementation during pregnancy (B =  −0.531) and paternal education level (B = −0.537) (Table 4). The risk factors associated with CHD were maternal adverse emotions during pregnancy (B = 0.975), maternal exposure to polluted water (B = 0.791), maternal severe vomiting (B = 0.648), maternal chronic diseases during pregnancy (B = 0.616) and paternal active smoking (B = 0.676) (Table 4).

**Table 4:** Results of Multivariate Conditional Logistic Analysis. Note: B = Beta; S.E = standard error; OR = odds ratio; 95% CI = 95% confidence interval.

Factor	B	S.E	P value	OR	95% CI
Maternal chronic diseases during pregnancy	0.616	0.308	0.046	1.852	1.012∼3.387
Maternal severe vomiting	0.648	0.297	0.029	1.911	1.067∼3.424
Maternal exposure to polluted water	0.791	0.294	0.007	2.205	1.240∼3.921
Maternal adverse emotions during pregnancy	0.975	0.169	0.000	2.650	1.903∼3.692
Maternal nutrition supplementation during pregnancy	−0.531	0.182	0.004	0.588	0.412∼0.841
Paternal education level	−0.537	0.188	0.004	0.585	0.405∼0.845
Paternal heavy metal exposure	0.688	0.352	0.050	1.990	0.999∼3.963
Paternal active smoking	0.676	0.189	0.000	1.967	1.357∼2.850
Constant	−3.643	0.819	0.000	0.026	

## Discussion

Understanding the associated parental non-hereditary teratogenic exposure factors for CHD in offspring is helpful in preventing birth defects. It is very important to use appropriate modelling strategies in studies of aetiologic association. To the best of our knowledge, this matched case-control study is the first to evaluate the associations between parental non-hereditary exposure factors and the risk of CHD in northeastern Sichuan. In addition, the questionnaire used in this study covered a wide range of exposures, which enabled us to explore multiple potential factors. However, selection bias can be a problem, as with other observational studies. In our study, affected foetuses that did not survive were excluded from the study population. If non-hereditary exposure factors increased the risk of severe birth defects and led to spontaneous and elective abortions, the associations between them would be underestimated, and collider stratification bias could introduce new confounders. Meanwhile, when different matching criteria are used, the statistical results may be different. In this matched case-control study, maternal adverse emotions during pregnancy, maternal exposure to polluted water, maternal severe vomiting, maternal chronic diseases during pregnancy and paternal active smoking increased the risk of the offspring developing CHD, whereas nutrition supplementation during pregnancy and paternal education level were significantly associated with a decreased risk of developing CHD.

1. Maternal adverse emotions during pregnancy. We observed that maternal adverse emotions during pregnancy (OR = 2.650, 95% CI: 1.903~3.692) were the most important factor in the occurrence of CHD. This association was also observed in the studies of LI Huixia *et al*.^18^. With the slowdown of Chinese economic growth, the major pressures of life and the gap between the rich and the poor, the fast pace of life and negative habits make psychological problems increasingly common^19^. As a special group, women have their own mental and psychological characteristics and are prone to depression, anxiety and other psychological problems^20^. Shaw, C *et al*.^21^ reported that stressful events during pregnancy are associated with cardiac malformations of the outflow tract, neural tube malformations and cleft lip. Xiaoqiang, Q *et al*.^22^ reported that psychological trauma or tension can stimulate the sympathetic adrenomedullin system and pituitary adrenocortical system to cause a series of physiological changes, thus increasing the risk of teratogenesis.
  Therefore, the influence of this mental and psychological factor on the occurrence of CHD is worthy of attention.
2. Maternal exposure to polluted water. Maternal exposure to polluted water (OR = 2.205, 95% CI: 1.240~3.921) was the second-most important factor in the occurrence of CHD. A considerable part of China’s drinking water resources come from groundwater. However, at the same time as social and economic development, groundwater resources have been overexploited by human beings, there is a serious shortage of resources, and groundwater also suffers a certain degree of pollution damage^23^. There are two sources of nitrate that pollute groundwater^23^. One is sewage and wastewater discharged from the surface, such as sewage from the urban septic tank, wastewater leaked from the sewage pipe, or rainwater leaching from the garbage heap. In the process of discharge, the polluted water from this source will infiltrate into the river, thus polluting the underground water resources^23^. This kind of pollution source has obvious characteristics of point source pollution^23^. Second, agricultural non-point sources pollute water, which causes the underground water resources to have excessive nitrate^23^. It is often necessary to apply nitrogen fertilizer in farming areas, so nitrogen fertilizer seeps into the ground; its content then becomes 12.5~45% nitrogen fertilizer^23^. Kim J *et al*.^24^ reported that pollutants in drinking water, such as trichloroethylene, tetrachloroethylene, dichloromethane, and benzene, were positively correlated with cardiac malformations. It was confirmed that trichloroacetic acid and trihalomethane in drinking water can increase the risk of ventricular septal defect (VSD)^25^. Dichloroethylene is the main cause of foetal cardiovascular malformation caused by polluted groundwater^26^. Therefore, we must pay attention to protecting the natural environment while developing the economy.
3. Paternal active smoking (OR = 1.967, 95% CI: 1.357~2.850) has been proven to be associated with an increased risk of CHD^27,28^. Bundhun PK *et al*.^29^ found that the mechanism of smoking may be related to the quantity and quality of sperm. It can also increase sperm DNA strand breaks and lead to sperm abnormalities^30^. If an abnormal sperm caused by smoking combines with an egg, it may lead to abnormal embryonic cardiac development^31^. China has the largest tobacco production and consumption in the world^32^. Nearly one million people die of smoking-related diseases every year, accounting for 12% of all deaths^32^. For the sake of our own health and that of our offspring, it is imperative to quit smoking.
4. Maternal severe vomiting (OR = 1.911, 95% CI: 1.067~3.424) was considered to be related to the occurrence of CHD in this study. Neither the cause nor the effect of severe vomiting during pregnancy is well understood^33^. Jiang Xuejing *et al*.^34^ reported that severe vomiting can lead to a rapid decline in body mass, malnutrition and dehydration in pregnant women, which can eventually have adverse effects on mothers and infants. The major limitation of this study is that severe vomiting, recorded from questionnaire records, was not rigorously defined, and misclassification in both directions may have occurred. However, it is unlikely that this limitation has strongly influenced our results. All the subjects included in the present analysis were controls who had offspring without CHD. Therefore, such a misclassification, if any, is likely to be no differential, which would bias the results towards the null.
5. Maternal chronic diseases during pregnancy (OR = 1.852, 95% CI: 1.012~3.387) were considered to be related to the occurrence of CHD in this study. Ereczkey, A *et al*.^35^ reported that certain chronic maternal diseases (i.e., epilepsy treated with carbamazepine, migraine, panic disorders, type I diabetes mellitus, chronic hypertension, paroxysmal supraventricular tachycardia) were found to be associated with a higher risk of specific types of CHD. However, this conclusion is rarely reported in the Chinese literature.
6. Maternal nutrition supplementation during pregnancy (OR = 0.588, 95% CI: 0.412~0.841) reduced the incidence of CHD. Qin C *et al*.^36^ confirmed that an effective measure to prevent CHD is the supplementation of the proper amount of multivitamins before and after pregnancy. Yuan SY *et al*.^37^ claimed that folic acid can also reduce the incidence of CHD, especially VSD in early pregnancy, which is consistent with the results of their study. This study suggests that pregnant women should maintain their own nutritional balance. To maintain balanced nutrition, pregnant women can take a balanced food nutrition spectrum or drugs supplemented with folic acid or multivitamins.
7. Paternal education level (OR = 0.585, 95% CI: 0.405~0.845) was another protective factor that reduced the incidence of CHD. The reason is that the father has a high level of education, has fewer manual workers, and is less likely to accept work with environmental exposure pollution, so the impact on his own sperm will be small^38^. At the same time, the cognitive ability of toxic and harmful substances is higher, which can prevent the exposure of pregnant women to such substances^10,38^.

The aetiology of CHD is complex, involving genetic factors, nonhereditary factors and the interaction of genetic factors and nonhereditary factors factors^1^. There are many non-hereditary factor-related factors, and as society evolves, those factors also change^25^. In the future, we will carry out a large-scale prospective survey of the population, examine the risk factors for CHD by differences in ethnic cultural backgrounds and medical and health care, quantify exposure indicators and detect and analyse the level of internal exposure.

## Conclusion

Augmenting maternal mental healthcare, improving the quality of drinking water, obtaining adequate nutrition, maintaining a healthy physical condition during pregnancy, enhancing parents’ knowledge level and maintaining a healthy lifestyle may lower the occurrence of CHD.

## Methods

### Research subjects

#### Sample size calculation

This study was designed as a case-control study with 1:1 frequency matching. The results of a meta-analysis by D. Jingmei suggest that adverse emotions during pregnancy were one of the strongest risk factors for CHD^39^. Maternal adverse emotions during pregnancy in the control group occurred in approximately 5% of the sample (expected OR = 3.0^39^), and the default selection was α = 0.05 (β = 0.1). A sample of 307 cases in the case group and the control group were calculated by PASS 11 software (Table 5). Therefore, the minimum sample size needed was 307.

**Table 5:** Matched Case-Control Power Analysis.

Numeric Results							
Power	Cases	Controls Per Case	Odds Ratio	Probability Exposed	Correlation	Alpha	Beta
	(N)	(M)	(OR)	(PO)	(Phi)		
0.90017	307	1	3.00	0.05000	0.20000	0.05000	0.09983

#### Sample source

This study included 367 children with CHD and 367 children without congenital malformations. All subjects aged 0 to 14 years old were recruited from the Affiliated Hospital of North Sichuan Medical College and Nanchong Central Hospital between March 2016 and November 2018.

#### Selection of the case group

All patients with CHD were consecutively enrolled children who were diagnosed by colour Doppler echocardiography.

The exclusion criteria were as follows: (1) Children with congenital malformations of other systems and other family genetic diseases; (2) children with myocarditis, cardiomyopathy and other cardiovascular diseases; (3) parents with conscious disorder, mental disorder, communication disorder, or poor memory of events during pregnancy; and (4) parents of children who did not agree to participate in the survey.

#### Selection of the control group

With respect to the admission criteria, controls without any congenital malformations were randomly selected among children who were undergoing physical disease treatment.

The exclusion criteria were as follows: (1) Children with other inherited familial diseases; (2) children with cardiovascular diseases such as myocarditis and cardiomyopathy; (3) parents with conscious disorder, mental disorder, communication disorder, or poor memory of events during pregnancy; and (4) parents who did not agree to participate in the survey.

### Research methodology

#### Survey content

The original data were obtained by means of questionnaires. Combined with the results of the meta-analysis of environmental risk factors of CHD in recent years, a questionnaire was developed. The content of the questionnaire included the following: (1) demographic characteristics: children’s physical age, gender, parents’ education levels^26^, permanent residence^40^ and parental ethnic origin^41,42^; (2) conditions of child: gestational age^43^ and birth weight^44^; (3) characteristics of parents: consanguineous marriage^3^, maternal age at pregnancy^26,45^, hazardous substance exposure history^10^ (electrical radiation, noise, heavy metals, pesticides, organic solvents, housing decorations, air pollution, water pollution), and parental lifestyle^31^ (smoking status^39^, consumption of alcohol^26^, drug dependence^46^ and sleeping status^47^); and (4) conditions of pregnancy: prenatal examination^48^, adverse childbearing history^26^, maternal chronic disease during pregnancy^49^, paternal chronic disease before pregnancy^49^, body mass index (BMI)^50,51^, abnormal pregnancy reactions^52^, nutrition status^10^, emotional status^53^, and medication history^54^.

#### Definition and assignment of variables

Adverse childbearing history refers to previous maternal history of spontaneous abortion and stillbirth and a history of birth defects. Chronic diseases refer to hepatitis, type 1 or 2 diabetes, hypertension, anaemia, obesity, rheumatism, tuberculosis, and malnutrition. Abnormal pregnancy reactions refer to threatened abortion, fever, headache, cough, sneeze, runny nose, infection of the female reproductive system, pregnancy complications, diarrhoea, fatigue and severe vomiting. Medication history refers to more than 1 day of taking the following drugs: Chinese herbal medicine, antibiotics, antipyretic analgesics, antihypertensive drugs, hypoglycaemic drugs, and medicine to prevent miscarriages. Electric radiation refers to daily contact with mobile phones, microwave ovens, induction cookers, and computers for more than 30 minutes. Exposure to heavy metals, pesticides and organic poisons refers to occupational or nonoccupational exposure to lead, mercury, cadmium, agricultural pesticides (insecticides, herbicides, rodenticide), benzene, paint, hair dye and other recognized harmful chemical agents during pregnancy. Noise refers to working or living in a noisy environment that causes maternal discomfort. The definition of air pollution is the existence of exhaust gas near the plant, a distance from the main traffic road of less than 50 m or work in the coal-fired power plant. Air pollution means that the air quality is determined by the local environmental protection department to be at the pollution level. Water pollution is defined as the pollution of drinking water or irrigation water for crops near the factory where the waste water is discharged or recognized by the local environmental protection department. Active smoking refers to daily smoking, while passive smoking refers to exposure to a smoking environment at home or at the workplace. Consumption of alcohol means drinking, which refers to drinking 250 ml of wine or the same amount of red wine and beer every day. Addictive drugs mainly refer to opioids and other drugs that individuals depend on psychologically or physiologically. Sleep disorder refers to difficulty falling asleep, light sleep, ease in waking up or waking up early. Adverse emotions during pregnancy refer to a bad mood, such as tension, injury, anxiety and depression during pregnancy at least once a month. Nutrition supplementation refers to the supplementation of high-quality protein and folic acid-based multivitamins and microelements during pregnancy. BMI was thin (≤18.4), normal (18.5–23.9), overweight (24.0–27.9), obese (≥28.0). Delivery was either premature (<37 weeks) or full-term (≥37 weeks). Birth weight was normal (2500 g), low (<2500 g) and macrosomic (>4000 g).

#### Data collection and management

One interviewer conducted face-to-face interviews of the participants’ birth parents using a standardized questionnaire. The paediatric cardiologists and interviewers were trained uniformly before the formal interview, and to further refine the format of the questionnaire and the skills of the investigators, a preliminary trial of the interview and questionnaire was conducted with the participation of the parents of a few patients from other medical institutions. All the participants’ birth parents were retrospectively interviewed in a similar manner during the period of the study. Informed consent was obtained from each participant.

#### Medical ethics

This study was approved by the ethics committee of the Affiliated Hospital of North Sichuan Medical College, and informed consent was obtained from the patients before the start of the study. We have read the Helsinki Declaration and have followed its guidelines in this study. Before each investigation, the purpose, significance, content, confidentiality principle and required time of the investigation shall be indicated to the interviewee or his or her guardian to ensure that the informed consent of his or her guardian is obtained, that the informed consent form shall be signed, and that the interviewee who does not agree with the investigation shall be respected and not investigated.

#### Quality control

Before the formal survey, the questionnaire was modified properly through a pre-survey, which made the survey more reasonable, feasible and effective. At the same time, the variables of the questionnaire were clearly defined and standardized.

### Statistical methods

#### Matching criterion

Using the SPSS 22.0 case-control matching module, the controls were matched to the cases at a rate of 1:1, according to the same gestational age of child (premature delivery or full-term), the maternal age of pregnancy (less than 1 year) and the same maternal racial/ethnic background. It is not clear whether there is a genetic relationship between maternal ethnic differences and children’s CHD^55,56^. The difference between gestational age and maternal gestational age may affect the duration of the foetus under the influence of non-genetic exposure factors. Therefore, in this study, the differences in maternal ethnicity, the gestational age of the child and maternal age during pregnancy were treated as potential confounders.

#### Data analysis

SPSS 22.0 software was used for statistical analysis. The matched case-control pairs were used to assess the potential factors for CHD by computing and comparing the exposure ratios. Potential demographic confounders were first screened through a univariate conditional logistic regression analysis. The variables that were identified as significant in the univariate analyses (P < 0.05) were entered into a multivariate conditional logistic regression model. For each categorical variable, a multivariate conditional logistic regression analysis was performed to compute the adjusted odds ratios (ORs) and 95% confidence intervals (CIs) and thus evaluate the variable’s association with CHD.

## Supplementary information

Supplementary Information.

## Data Availability

The datasets generated during and/or analyzed during the current study are available from the corresponding author on reasonable request.
