# Supplementary material for: Parental non-hereditary teratogenic exposure factors on the occurrence of congenital heart disease in the offspring in the northeastern Sichuan, China
Source: Sci Rep. 2020 Mar 3;10:3905. doi: 10.1038/s41598-020-60798-6 (PMC7054293; doi:10.1038/s41598-020-60798-6)
Supplement: Supplementary file 1 — Supplementary Information. [file 41598_2020_60798_MOESM1_ESM.docx]

**Parental non-hereditary teratogenic exposure factors on the occurrence of congenital heart disease in the offspring in the northeastern Sichuan, China**

Yun Liang^1, +^, Xingsheng HU^2, +^, Xiaoqin Li^3, +^, Bing Wen^4^, Liang Wang^1^, Cheng Wang^1*^

^1^Department of Pediatric Surgery, Affiliate Hospital of North Sichuan Medical College, Nanchong, 637000, P.R. China. ^2^Department of Oncology, the Second Xiangya Hospital of Central South University, Changsha, Hunan, 41011, P.R. China. ^3^Department of Nursing, Affiliate Hospital of North Sichuan Medical College, Nanchong, 637000, P.R. China. ^4^Department of Cardiothoracic Surgery, Nanchong Central Hospital, Nanchong, 637000, P.R. China.

**^+^****These authors contributed equally: Yun Liang, Xingsheng HU, Xiaoqin Li.**

***Correspondence:**

Cheng Wang, MD

Department of Pediatric Surgery

Affiliate Hospital of North Sichuan Medical College

No 63 Wenhua Road, Nanchong, 637000, P.R. China

TEL: +(86)18227398290.

Email: [178399881@qq.com](mailto:178399881@qq.com), [361889874@qq.com](mailto:361889874@qq.com);

**Abstract words:** 200 words

**Text words:** 3200 words

**Supplementary materials**

| **Research factors** | **Controls**  **N=322** | | **Cases**  **N=322** | | **P value** |
| --- | --- | --- | --- | --- | --- |
|  | **N** | **%** | **N** | **%** |  |
| **Maternal factors** |  |  |  |  |  |
| **Permanent residence** |  |  |  |  |  |
| Downtown | **244** | **75.8** | **232** | **72** | **0.282** |
| Rural area | **78** | **24.2** | **90** | **28** |  |
| **Education level*** |  |  |  |  | **0.027** |
| Primary or junior high school | **43** | **13.4** | **45** | **14** |  |
| Senior high school | **77** | **23.9** | **106** | **32.9** |  |
| Master’s degree or higher college | **202** | **62.7** | **171** | **53.1** |  |
| **Adverse childbearing history*** |  |  |  |  | **0.007** |
| None | **295** | **91.6** | **273** | **84.8** |  |
| Yes | **27** | **8.4** | **49** | **15.2** |  |
| **Family history of congenital heart disease*** |  |  |  |  | **0.033** |
| None | **285** | **88.5** | **266** | **56** |  |
| Yes | **37** | **11.5** | **82.6** | **17.4** |  |
| **BMI** |  |  |  |  | **0.132** |
| Thin (≤18.4) | **36** | **11.2** | **48** | **14.9** |  |
| Normal (18.5-23.9) | **189** | **58.7** | **163** | **50.6** |  |
| Overweight (24.0-27.9) | **89** | **27.6** | **97** | **30.1** |  |
| Obese (≥28.0) | **8** | **2.5** | **14** | **4.3** |  |
| **Chronic diseases during pregnancy*** |  |  |  |  | **0.005** |
| None | **301** | **93.5** | **280** | **87** |  |
| Yes | **21** | **6.5** | **42** | **13** |  |
| **Threatened abortion** |  |  |  |  | **0.896** |
| None | **290** | **90.1** | **289** | **89.8** |  |
| Yes | **32** | **9.9** | **33** | **10.2** |  |
| **Fever*** |  |  |  |  | **0.001** |
| None | **299** | **92.9** | **273** | **84.8** |  |
| Yes | **23** | **7.1** | **49** | **15.2** |  |
| **Headache** |  |  |  |  | **0.089** |
| None | **277** | **86.0** | **261** | **81.1** |  |
| Yes | **45** | **14.0** | **61** | **18.9** |  |
| **Cough*** |  |  |  |  | **0.007** |
| None | **267** | **82.9** | **239** | **74.2** |  |
| Yes | **55** | **17.1** | **83** | **25.8** |  |
| **Sneeze**  **and runny nose*** |  |  |  |  | **0.003** |
| None | **251** | **78.0** | **218** | **67.7** |  |
| Yes | **71** | **22.0** | **104** | **32.3** |  |
| **Infection of female reproductive system*** |  |  |  |  | **0.005** |
| None | **313** | **97.2** | **297** | **92.3** |  |
| Yes | **9** | **2.8** | **25** | **7.8** |  |
| **Pregnancy complications*** |  |  |  |  | **0.01** |
| None | **272** | **84.5** | **246** | **76.4** |  |
| Yes | **50** | **15.5** | **76** | **23.6** |  |
| **Diarrhoea*** |  |  |  |  | **0.003** |
| None | **312** | **96.9** | **294** | **91.3** |  |
| Yes | **10** | **3.1** | **28** | **8.7** |  |
| **Fatigue*** |  |  |  |  | **0.049** |
| None | **235** | **73.0** | **212** | **65.8** |  |
| Yes | **87** | **27.0** | **110** | **34.2** |  |
| **Severe vomiting*** |  |  |  |  | **0.001** |
| None | **300** | **93.2** | **274** | **85.1** |  |
| Yes | **22** | **6.8** | **48** | **14.9** |  |
| **Chinese herbal medicine*** |  |  |  |  | **0.005** |
| None | **295** | **91.6** | **272** | **84.5** |  |
| Yes | **27** | **8.4** | **50** | **15.5** |  |
| **Antibiotic** |  |  |  |  | **0.032** |
| None | **280** | **87.0** | **260** | **80.7** |  |
| Yes | **42** | **13.0** | **62** | **19.3** |  |
| **Antipyretic analgesics*** |  |  |  |  | **0.005** |
| None | **268** | **83.2** | **239** | **74.2** |  |
| Yes | **54** | **16.8** | **83** | **25.8** |  |
| **Antihypertensive drugs** |  |  |  |  | **0.056** |
| None | **315** | **97.8** | **306** | **95.0** |  |
| Yes | **7** | **2.2** | **16** | **5.0** |  |
| **Hypoglycaemic drugs** |  |  |  |  | **0.103** |
| None | **316** | **98.1** | **309** | **96.0** |  |
| Yes | **6** | **1.9** | **13** | **4.0** |  |
| **Medicine to prevent miscarriages *** |  |  |  |  | **0.021** |
| None | **297** | **92.2** | **279** | **86.6** |  |
| Yes | **25** | **7.8** | **43** | **13.4** |  |
| **Electrical radiation*** |  |  |  |  | **0.039** |
| ≤1 item | **101** | **31.4** | **85** | **26.4** |  |
| 2 items | **195** | **60.6** | **192** | **59.6** |  |
| ≥3 items | **26** | **8.1** | **45** | **14.0** |  |
| **Noise** |  |  |  |  | **0.166** |
| None | **292** | **90.7** | **281** | **87.3** |  |
| Yes | **30** | **9.3** | **41** | **12.7** |  |
| **Heavy metals*** |  |  |  |  | **0.021** |
| None | **304** | **94.4** | **288** | **89.4** |  |
| Yes | **18** | **5.6** | **34** | **10.6** |  |
| **Pesticides*** |  |  |  |  | **0.005** |
| None | **313** | **97.2** | **297** | **92.2** |  |
| Yes | **9** | **2.8** | **25** | **7.8** |  |
| **Organic solvents** |  |  |  |  | **0.188** |
| None | **279** | **86.6** | **267** | **82.9** |  |
| Yes | **43** | **13.4** | **55** | **17.1** |  |
| **Housing decorations** |  |  |  |  | **0.239** |
| None | **301** | **93.5** | **293** | **91.0** |  |
| Yes | **21** | **6.5** | **29** | **9.0** |  |
| **Air pollution** |  |  |  |  | **0.187** |
| None | **269** | **83.5** | **256** | **79.5** |  |
| Yes | **53** | **16.5** | **66** | **20.5** |  |
| **Water pollution*** |  |  |  |  | **0.000** |
| None | **300** | **93.2** | **265** | **82.3** |  |
| Yes | **22** | **6.8** | **57** | **17.7** |  |
| **Active smoking** |  |  |  |  | **0.057** |
| None | **290** | **90.1** | **276** | **85.7** |  |
| ≤20 cigarettes/day | **30** | **9.3** | **46** | **14.3** |  |
| >20 cigarettes/day | **2** | **0.6** | **0** | **0.0** |  |
| **Passive smoking*** |  |  |  |  | **0.001** |
| None | **269** | **83.5** | **234** | **72.7** |  |
| Yes | **53** | **16.5** | **88** | **27.3** |  |
| **Drinking*** |  |  |  |  | **0.006** |
| None | **293** | **91.0** | **266** | **82.6** |  |
| Yes | **29** | **9.0** | **56** | **22.4** |  |
| **Addictive drugs** |  |  |  |  | **0.461** |
| None | **315** | **97.8** | **312** | **96.9** |  |
| Yes | **7** | **2.2** | **10** | **3.1** |  |
| **Sleep disorder** |  |  |  |  | **0.066** |
| None | **305** | **94.7** | **293** | **91.0** |  |
| Yes | **17** | **5.3** | **29** | **9.0** |  |
| **Adverse emotions during pregnancy*** |  |  |  |  | **0.000** |
| None | **243** | **75.5** | **156** | **48.4** |  |
| Yes | **79** | **24.5** | **166** | **51.6** |  |
| **Nutrition supplementation*** |  |  |  |  | **0.000** |
| None | **179** | **55.6** | **226** | **70.2** |  |
| Yes | **143** | **44.4** | **96** | **29.8** |  |
| **Periodic prenatal examination** |  |  |  |  | **0.069** |
| None | **138** | **42.9** | **161** | **50.0** |  |
| Yes | **184** | **57.1** | **161** | **50.0** |  |
| **Paternal factors** |  |  |  |  |  |
| **Permanent residence *** |  |  |  |  | **0.007** |
| Downtown | **272** | **84.5** | **245** | **76.1** |  |
| rural area | **50** | **15.5** | **77** | **23.9** |  |
| **Education level*** |  |  |  |  | **0.000** |
| High school or less | **80** | **24.8** | **137** | **42.5** |  |
| University or above | **242** | **75.2** | **185** | **57.5** |  |
| **Chronic diseases** |  |  |  |  | **0.076** |
| None | **307** | **95.3** | **296** | **91.9** |  |
| Yes | **15** | **4.7** | **26** | **8.1** |  |
| **Electrical radiation** |  |  |  |  | **0.334** |
| ≤2items | **287** | **89.1** | **279** | **86.6** |  |
| ≥3items | **35** | **10.9** | **43** | **13.4** |  |
| **Noise** |  |  |  |  | **0.222** |
| None | **268** | **89.8** | **279** | **86.6** |  |
| Yes | **33** | **10.2** | **43** | **13.4** |  |
| **Heavy metals*** |  |  |  |  | **0.001** |
| None | **307** | **95.3** | **284** | **88.2** |  |
| Yes | **15** | **4.7** | **38** | **11.8** |  |
| **Pesticides*** |  |  |  |  | **0.011** |
| None | **311** | **96.6** | **296** | **91.9** |  |
| Yes | **11** | **3.4** | **26** | **8.1** |  |
| **Organic solvents** |  |  |  |  | **0.517** |
| None | **274** | **85.1** | **268** | **83.2** |  |
| Yes | **48** | **14.9** | **54** | **16.8** |  |
| **Housing Decorations** |  |  |  |  | **0.338** |
| None | **304** | **94.4** | **298** | **92.5** |  |
| Yes | **18** | **5.6** | **24** | **7.5** |  |
| **Air pollution** |  |  |  |  | **0.749** |
| None | **271** | **84.2** | **268** | **83.2** |  |
| Yes | **51** | **15.8** | **54** | **16.8** |  |
| **Water pollution** |  |  |  |  | **0.809** |
| None | **284** | **88.2** | **282** | **87.6** |  |
| Yes | **38** | **11.8** | **40** | **12.4** |  |
| **Active smoking*** |  |  |  |  | **0.000** |
| None | **247** | **76.7** | **186** | **57.8** |  |
| Yes | **75** | **23.3** | **136** | **42.2** |  |
| **Passive smoking*** |  |  |  |  | **0.000** |
| None | **254** | **78.9** | **209** | **64.9** |  |
| Yes | **68** | **21.1** | **113** | **35.1** |  |
| **Drinking*** |  |  |  |  | **0.001** |
| None | **257** | **79.8** | **219** | **68.0** |  |
| Yes | **65** | **20.2** | **103** | **32.0** |  |
| **Addictive drugs** |  |  |  |  | **0.056** |
| None | **317** | **98.4** | **309** | **96.0** |  |
| Yes | **5** | **1.6** | **13** | **4.0** |  |
| **Sleep disorder*** |  |  |  |  | **0.003** |
| None | **298** | **92.5** | **274** | **85.1** |  |
| Yes | **24** | **7.5** | **48** | **14.9** |  |

**Table 1** Univariate analysis of research factors among cases and controls. Note: BMI=Body mass index;

*=Statistically significant after univariate analysis with a test criterion of 0.05
